# Microbiome Dysbiosis Is Associated with Castration Resistance and Cancer Stemness in Metastatic Prostate Cancer

**DOI:** 10.3390/ijms25063291

**Published:** 2024-03-14

**Authors:** Matthew Uzelac, Ruomin Xin, Weg M. Ongkeko

**Affiliations:** 1 Department of Surgery, Division of Otolaryngology-Head and Neck Surgery, University of California San Diego, La Jolla, CA 92093, USA; 2 Research Service, VA San Diego Healthcare System, San Diego, CA 92161, USA

**Keywords:** prostate cancer, castration-resistant prostate cancer, microbiome, cancer stem cells

## Abstract

Prostate cancer is the second leading cause of death in males in America, with advanced prostate cancers exhibiting a 5-year survival rate of only 32%. Castration resistance often develops during the course of treatment, but its pathogenesis is poorly understood. This study explores the human microbiome for its implications in castration resistance and metastasis in prostate cancer. RNA sequencing data were downloaded for the bone and soft tissue biopsies of patients with metastatic castration-resistant prostate cancer. These included both metastatic and adjacent normal biopsies. These sequences were mapped to bacterial sequences, yielding species-level counts. A vast majority of species were found to be significantly underabundant in the CRPC samples. Of these, numerous were found to correlate with the expression of known markers of castration resistance, including AR, PI3K, and AKT. Castration resistance-associated signaling pathways were also enriched with these species, including PI3K-AKT signaling and endocrine resistance. For their implications in cancer aggression and metastasis, cancer stem cell markers were further explored for a relation to these species. EGFR and SLC3A2 were widely downregulated, with a greater abundance of most species. Our results suggest that the microbiome is heavily associated with castration resistance and stemness in prostate cancer. By considering the microbiome’s importance in these factors, we may better understand the highly aggressive and highly invasive nature of castration-resistant prostate cancer, allowing for the needed improvements in the treatment of this disease.

## 1. Introduction

Prostate cancer is the second leading cause of death in males in America [1,2]. It is estimated that roughly one in eight men will be diagnosed with prostate cancer in their lifetime [1,2]. This type of cancer is classified as localized, regional, or distant based on the degree by which it has metastasized to other bodily sites. Bone metastases are the most common, followed by lymph node and liver metastases [3]. Localized and regional prostate cancers are rarely fatal, though distant prostate cancers exhibit a 5-year survival rate of only 32% [1]. Understanding the factors that promote the acquisition of this more aggressive stage is crucial to improving these patients’ survival.

The growth and proliferation of prostate cancer is known to be heavily mediated by testosterone signaling [4]. In a healthy individual, testosterone is capable of binding to and activating the androgen receptor (AR) protein, which then stimulates the production of secretory proteins in the prostate [5,6]. Through mechanisms not well understood, specific mutations in the genes of the AR signaling pathway are known to ultimately promote the growth and proliferation of prostate cancer [5,6]. As such, androgen deprivation therapies are commonly implemented as treatments for high-risk prostate cancers [7]. These largely include medical and surgical castrations [7]. Nonetheless, these therapies yield fairly poor survival rates, as the patients who undergo castration likely have more advanced diseases [7,8]. Many patients develop castration resistance (CR), which is defined by the sustained growth of a cancer despite serum testosterone levels being at or below the level expected with castration. The mechanisms by which prostate cancers acquire this resistance are well characterized, with AR signaling being integral to many of them [9]. These involve mutations in AR, mutations in AR coactivators and corepressors, androgen-independent activation of AR, and alternate means of androgen biosynthesis [9]. Several other mechanisms of resistance have been explored, including the dysregulation of the lipid metabolism and the mevalonate pathway [10]. For the patients who develop CR, AR signaling inhibitors are often prescribed as an adjuvant treatment to androgen deprivation therapy [11,12,13,14,15,16]. However, many patients remain insensitive to these as well [17], with few treatment options available thereafter. The median survival length for non-metastatic castration-resistant prostate cancer (CRPC) cases is estimated to be 30.3 months and that for metastatic CRPC cases is only 13.3 months. Understanding the factors that influence the acquisition of CR in prostate cancers may significantly improve our ability to treat these diseases. Understanding the influences of metastasis in CRPC may prove doubly useful.

Numerous genetic factors have been identified for their implications in CRPC, primarily comprising mutations in the genes of the AR signaling pathway [9]. Metastatic contributors have been identified too, and they include the loss of PTEN, aberrations in the PI3K-AKT signaling pathway, and the acquisition of DNA repair defects [18]. The degree of stemness observed in a cancer is also known to influence CR and metastasis [19,20,21]. Cancer stem cells (CSC) are thought to compose only about 1% of a tumor’s mass, though they are crucial in the tumor’s growth and proliferation [22]. CSCs are believed to originate from epithelial cells through a process known as epithelial–mesenchymal transition (EMT) [23]. In this process, malignant epithelial cells gain mesenchymal-like traits and become highly invasive in doing so [24]. The formation of CSCs through the process of EMT provides a tumor with a high capacity for colonization, ultimately promoting the cancer’s metastasis [19,20,21].

The influences of epigenetic factors in CRPC are less explored, though the human microbiome may be highly relevant to CRPC The microbiome is a collection of bacterial and fungal microorganisms that reside largely in the gastrointestinal system [25]. Over recent decades, the microbiome has seen increased implication in human diseases, including inflammatory bowel disease and diabetes, among others [26,27]. Moreover, the microbiome has been shown to influence an array of biological processes and is believed to act through metabolite-mediated immune modulation [28,29]. Studies have also investigated the microbiome for its role in cancer, especially colorectal cancers [30,31,32,33]. Less is known of the microbiome’s influence on other cancer types, though studies have demonstrated the importance of the microbiome in prostate cancers [34,35]. Specific dysbioses of the gut microbiome have been identified between castration-sensitive (CS) and CR prostate cancers [35]. Moreover, antibiotic therapies and fecal transplants in mouse models have demonstrated the gut microbiome’s ability to modulate the effectiveness of androgen deprivation therapy [36]. The mechanisms behind these relations are less understood.

Hence, this study attempts to characterize microbiome dysbioses for correlations to both CR and cancer stemness. RNA sequencing data were downloaded for the bone and soft tissue biopsies of patients with metastatic CRPC across two studies: phs000915 (*n* = 147) and phs001141 (*n* = 143). Whole-exome sequencing data were downloaded for the adjacent normal biopsies from the above study, phs000915 (*n* = 84). These sequences were mapped to bacterial sequences, yielding species-level abundance counts for each sample. We identified numerous species that were differentially abundant between the CRPC and the normal samples. We also identified correlations of these species to known transcriptional markers of CR and cancer stemness. Further, we observed enrichment of the AR, PI3K-AKT, and endocrine resistance signaling pathways with respect to these species’ abundance. We propose that the human microbiome is heavily associated with CR and metastasis in prostate cancer. Through this investigation, we may better understand the pathology of metastatic CRPC, creating new avenues of research for the treatment of this disease.

## 2. Results

### 2.1. Cross-Study Normalization and Contamination Correction

The samples of two separate datasets were analyzed in this study. In order to account for innate differences in the sample collection and sequencing procedures used by these datasets, cumulative sum scaling was performed as a means of normalization (see Section 4). PCoA was conducted to demonstrate the effectiveness of this technique. In this analysis, the abundance counts of all the species in a sample were reduced to unitless and arbitrary dimensions. In these dimensions, the samples of a closer proximity had greater similarity in their abundance profiles. The samples were analyzed both before (Supplementary Materials Figure S1A) and after (Supplementary Materials Figure S1B) the normalization procedures were performed. After normalization, the samples were observed to be of a closer proximity, with fewer outliers present. This served to confirm the compatibility of the chosen samples for further analyses. Cumulative sum scaling was similarly performed on the samples’ gene expression counts.

In tissue extraction and sequencing procedures, there remains the possibility for contaminant species to be introduced into a sample [37]. Contamination correction was performed to identify and exclude these species (see Section 4). To visualize the phylogenic division of these species, they were grouped by class of phylum and plotted in a phylogenic tree (Supplementary Materials, Figure S1C). The bone metastases were found to contain the greatest number of contaminant species, followed by the liver and lymph node metastases.

### 2.2. Differential Abundance Analysis

**Figure 1:**
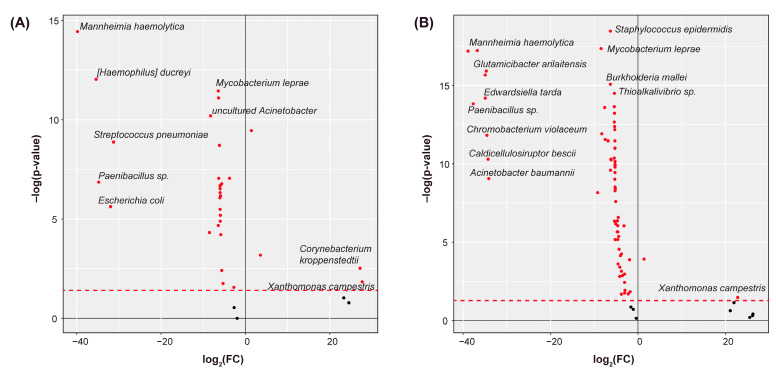

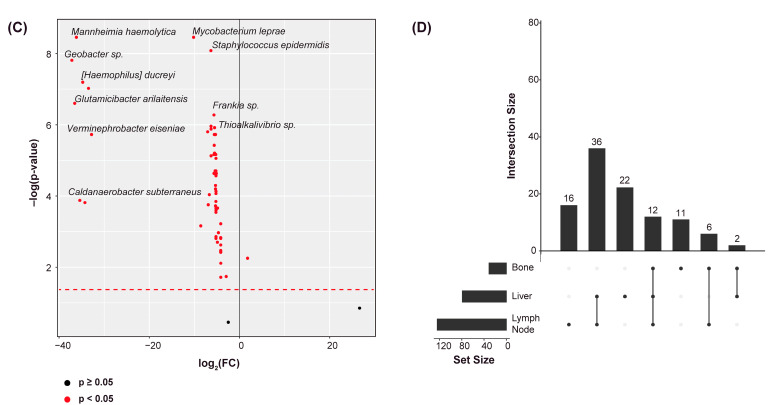
Differentially abundant species. Volcano plots showing the species that were differentially abundant between the CRPC tumor and adjacent normal tissue samples for the (**A**) bone, (**B**) lymph node, and (**C**) liver cohorts. Fold-changes (FC) indicate the change in each species’ abundance between the tumor and the normal samples. Heights indicate significance. (**D**) UpSet plot showing the number of differentially abundant species that were common to each site of metastasis. Twelve species were common to all sites.

We analyzed the remaining species to determine if any were differentially abundant between the CRPC tumor and the normal tissue samples. Using the Kruskal–Wallis test (*p* < 0.05), we identified 31 differentially abundant species in the bone cohort (Figure 1A), 70 species in the lymph node cohort (Figure 1B), and 65 species in the liver cohort (Figure 1C). In all the cohorts, a vast majority of the species were of significantly lesser abundance in the CRPC samples. This is consistent with the notion that castration causes ablation of the human microbiome [38,39]. Twelve species were common to all of these cohorts, including several strains of *Escherichia coli*, *Acinetobacter* sp., and *Mycobacterium leprae* (Figure 1D). We chose to further analyze only the species that were differentially abundant within each respective cohort. This served to demonstrate the relationship between CRPC-related microbiome dysbiosis and CRPC pathophysiology.

### 2.3. Species Abundance Correlates with Castration Resistance Marker Expression

**Figure 2:**
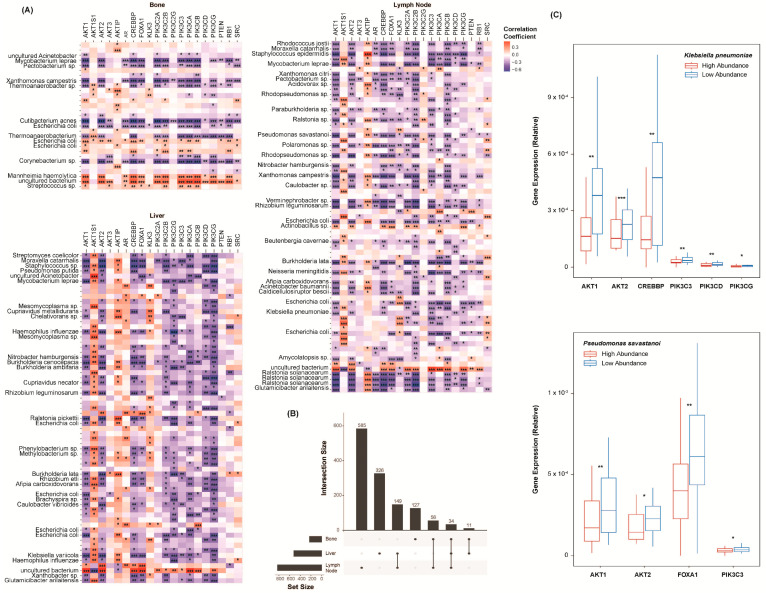
Species castration resistance marker correlations. (**A**) Heatmaps showing species’ correlations to CR marker expression, grouped by the site of metastasis. Colors indicate the strength of the correlations. (**B**) UpSet plot showing the number of species–marker correlations common to each site of metastasis. Thirty-four correlations were common to all the sites. (**C**) Box plots showing the expression of AKT1, AKT2, CREBBP, PIK3C3, PIK3CD, PIK3CG, and FOXA1 with respect to the abundance of *Klebsiella pneumoniae* and *Pseudomonas savastanoi*. The samples were grouped based on their relation to the median abundance of each species. The expression values are relative due to the method of cross-study normalization employed (see Section 4). * *p* < 0.05, ** *p* < 0.01, and *** *p* < 0.001.

A list of markers known to be implicated in CR at a transcriptional level was collected from the literature [18]. Among others, it includes AR, PTEN, several PI3K family genes, and several AKT family genes (Supplementary Materials Table S1). Spearman’s correlations were used to assess the relationship between each marker and each species. Again, only the species found to be differentially abundant between the CRPC and the normal samples were included. The correlations of all the species to these markers are included in the Supplemental Materials (Figure S2). In all the cohorts, a considerable number of species were significantly correlated to the CR markers (Figure 2A). Among others, a greater abundance of *Staphylococcus epidermidis* corresponded to an increase in the expression of all but a few of the markers. The lesser abundance of *Streptococcus pneumoniae* and *Mannheimia haemolytica* also corresponded to an increase in the markers’ expression. The overlap in the species–marker correlations between each cohort was further plotted (Figure 2B). Thirty-four significant correlations were common to all the sites of metastasis. We next simplified the abundance values of each species into binary classifications, either “high abundance” or “low abundance”, based on each sample’s relation to the median abundance of that species. For only the significant correlations above, the expression counts of several markers were plotted with respect to the abundance of *Klebsiella pneumoniae* and *Pseudomonas savastanoi* (Figure 2C). The lesser abundance of these species corresponded to a significantly greater expression of the AKT and PI3K family genes.

### 2.4. Castration Resistance Pathway Enrichment

Select KEGG gene sets were chosen to model the cellular pathways of CR (Supplementary Materials Table S1). The prostate cancer pathway was chosen to model the AR signaling cascade, containing many of its component genes, such as AR, CREBBP, and PTEN [18]. PI3K-AKT signaling was also chosen for analysis, as it is known to be upregulated in patients with CRPC [18,40]. The activation of this pathway has been shown to contribute to the growth and proliferation of prostate tumors in preclinical models [41]. The endocrine resistance pathway was chosen to model the resistance to androgen deprivation therapy. This gene set contains many of the genes that have been implicated in CR, including many of the PIK family genes [9,18].

**Figure 3:**
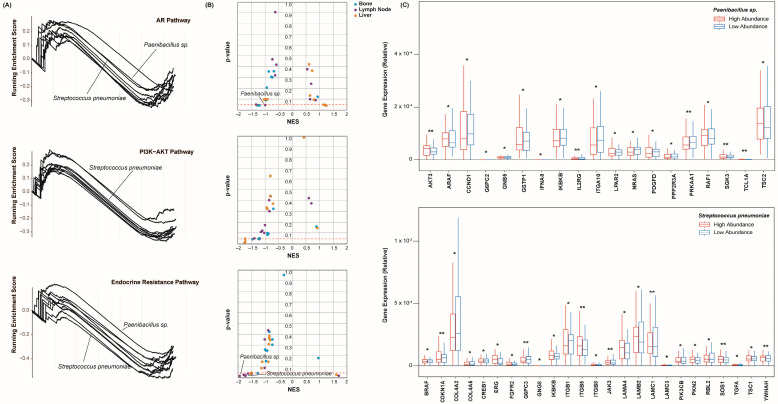
Species-associated enrichment of castration resistance pathways. (**A**) Enrichment plots of the AR (**top**), PI3K-AKT (**middle**), and endocrine resistance (**bottom**) signaling pathways. Each line represents a species. The peak of each curve indicates the total enrichment score of the pathway with respect to each species’ abundance. Only the twenty species of the lymph node cohort with the greatest number of significant correlations to the above CR markers are shown. (**B**) Scatter plots showing the enrichment of the AR (**top**), PI3K-AKT (**middle**), and endocrine resistance (**bottom**) signaling pathways with respect to the species’ abundances. The points represent the species. The nominal enrichment scores (NES) describe the direction and strength of the enrichment, and the heights signify significance. The points are colored according to the metastatic sites. (**C**) Box plots showing the expression of several genes of the AR signaling pathway with respect to the abundance of the *Paenibacillus* sp. and *Streptococcus pneumoniae*. The samples were grouped based on their relation to the median abundance of each species. The expression values are relative due to the method of cross-study normalization employed (see Section 4). * *p* < 0.05, and ** *p* < 0.01.

A gene set enrichment analysis was conducted to assess the extent to which each of these pathways was enriched with respect to each species’ abundance. For the lymph node cohort, we created enrichment plots to display the enrichment score of these pathways in each species (Figure 3A). Across the bone, lymph node, and liver cohorts, we identified a total of 4116 species to be present intratumorally. Due to this size, only the twenty species with the greatest number of significant correlations to the above CR markers are shown in the plots below. Given their correlations to the CR markers, we suspected that these species would also have the strongest correlation to these CR pathways. The peak of each curve indicates the total enrichment score of the pathway. Of all the cohorts, these pathways were found to be negatively enriched with respect to most species (Figure 3B), meaning that a lesser abundance of these species corresponded to an increase in AR signaling, PIK3-AKT signaling, and endocrine resistance. Of note, only a handful of species yielded an FDR < 0.20. The individual genes of each pathway were further assessed for correlation to these species’ abundances. This served to determine the significance by which each of the pathways’ components was enriched. The abundance values of each species were simplified to “high” or “low” classifications, as performed above. The expression counts of several genes of the AR signaling pathway were plotted with respect to the abundance of the *Paenibacillus* sp. and *Streptococcus pneumoniae* in the bone cohort (Figure 3C). A considerable number of genes were significantly dysregulated with the greater abundance of these species, including several PIK and AKT family genes. Ultimately, these species and others appear to closely reflect the analyzed pathways. Whether these are mechanistically linked is unclear, as is the direction of their potential regulation.

### 2.5. Species Abundance Correlates with Cancer Stem Cell Marker Expression

**Figure 4:**
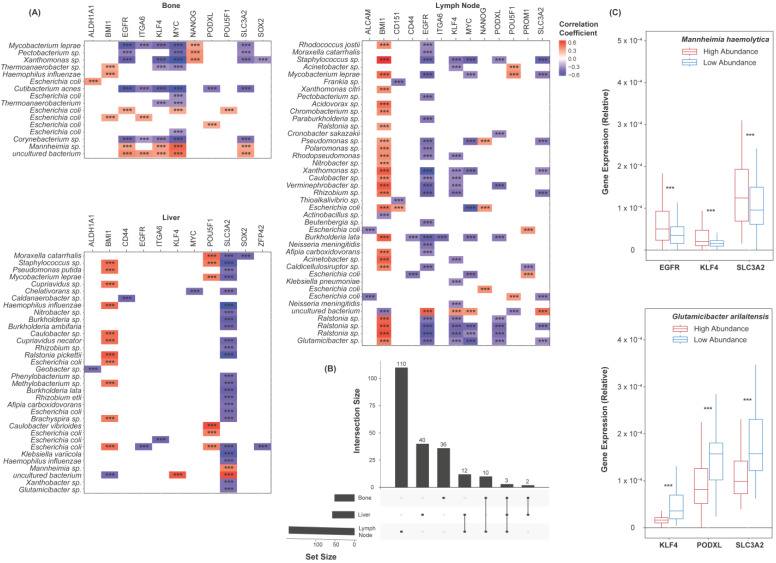
Species–cancer stem cell marker correlations. (**A**) Heatmaps showing species’ correlations to CSC markers’ expression, grouped by the site of metastasis. Colors indicate the correlation coefficients. (**B**) UpSet plot showing the number of species–marker correlations common to each site of metastasis. Three correlations were common to all sites. (**C**) Box plots showing the expression of EGFR, KLF4, SLC3A2, and PODXL with respect to the abundance of *Mannheimia haemolytica* and *Glutamicibacter arilaitensis*. The samples were grouped based on their relation to the median abundance of each species. The expression values are relative due to the method of cross-study normalization employed (see Section 4). *** *p* < 0.001.

Cancer stemness is known to be heavily implicated in CR and metastasis [19,20,21]. CSCs are thought to have a high capacity for migration and colonization and are widely believed to be the basis of metastasis across cancer types [19,20,21]. To assess the microbiome’s relation to these factors, a list of transcriptional CSC markers was first collected from the literature [42,43,44,45]. Among others, this list includes CD44, SOX2, and NANOG (Supplementary Materials Table S1). Spearman’s correlations were used to assess the relationship between each marker and each species. Only the species found to be differentially abundant between the CRPC and the normal samples were included. The correlations of all the species to these markers are included in the Supplemental Materials (Figure S3). Of all the cohorts, select markers appeared to be broadly upregulated or broadly downregulated with respect to these species (Figure 4A). MYC, EGFR, and SLC3A2 expression was negatively correlated to the abundance of all but a few species. BMI1 was widely overexpressed with a greater abundance of the above species. It is unclear whether these genes are linked to the microbiome mechanistically, though the consistency in the correlations’ directions should be noted. The overlap in the species–marker correlations between each cohort was further plotted (Figure 4B). Only three significant correlations were common to all the sites of metastasis. We again simplified the abundance values of each species into binary classifications, being either “high abundance” or “low abundance”. The expression counts of EGFR, KLF4, SLC3A2, and PODXL were plotted with respect to the abundance of *Mannheimia haemolytica* and *Glutamicibacter arilaitensis* (Figure 4C). A lesser abundance of *Glutamicibacter arilaitensis* corresponded to a significantly greater expression of these markers, while a lesser abundance of *Mannheimia haemolytica* corresponded to significantly a lesser expression of these markers.

### 2.6. Epithelial–Mesenchymal Transition and Pluripotency Regulation Pathway Enrichment

CSC formation is believed to be dependent on the invasion of epithelial cells through the process of EMT [25]. The microbiome’s relevance to these processes has been demonstrated [46,47], though not with regard to CR or metastasis in prostate cancer. Select KEGG gene sets were first chosen to model EMT and stemness (Supplementary Materials Table S1). The adherens junction pathway contains many of the genes involved in EMT and was chosen to model this process [48]. The Signaling Pathways Regulating Plurioptentcy of Stem Cells was chosen to model stemness.

**Figure 5:**
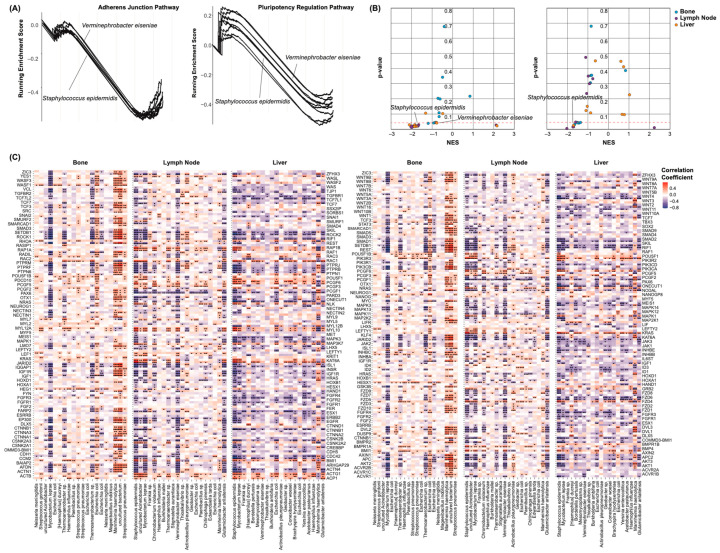
Species-associated enrichment of cancer stemness pathways. (**A**) Enrichment plots of the EMT (**left**) and pluripotency regulation (**right**) signaling pathways. Each line represents a species. The peak of each curve indicates the total enrichment score of the pathway with respect to each species’ abundance. Only the twenty species of the lymph node cohort with the greatest number of significant correlations to the above CSC markers are shown. (**B**) Scatter plots showing the enrichment of the EMT (**left**) and pluripotency regulation (**right**) pathways with respect to species’ abundances. The points represent the species. NESs describe the direction and strength of the enrichment, and the heights signify significance. The points are colored according to the metastatic sites. (**C**) Heatmaps showing the correlation of each species to the expression of the component genes of the EMT (**left**) and pluripotency regulation (**right**) pathways, grouped by the site of metastasis. The colors indicate the correlation coefficients. Only the twenty species of each cohort with the greatest number of significant correlations to the above CSC markers are shown. * *p* < 0.05, ** *p* < 0.01, and *** *p* < 0.001.

A gene set enrichment analysis was again conducted to assess these pathways for enrichment with respect to each species’ abundance. For the lymph node cohort, we created enrichment plots to display the enrichment score of these pathways with each species (Figure 5A). Only the twenty species with the greatest number of significant correlations to the above CSC markers are shown. Of all the species, we suspected that these would also correlate the most strongly to the EMT and pluripotency pathways. Similar to the AR signaling, PI3K-AKT signaling, and endocrine resistance pathways above, these pathways were negatively enriched with respect to all but a few species (Figure 5B). This may indicate that EMT and stemness are greater in patients with a lack of microbial diversity. Only a handful of species yielded an FDR < 0.20. The individual components of each pathway were assessed for correlation to these species’ abundances. Spearman’s correlations were computed between each gene’s expression and each species’ abundance. The resultant correlation coefficients and test statistics were plotted for all the cohorts (Figure 5C). Only the twenty species with the greatest number of significant correlations to the above CSC markers were analyzed. In each cohort, several species appeared to correlate with a majority of these genes in a consistent direction. A decreased abundance of *Mycobacterium leprae*, for instance, correlated to an increased expression of all but a few genes of the EMT and pluripotency pathways. Moreover, many genes were common to both of these pathways, perhaps explaining why these results appeared to mirror one another closely.

Due to the microbiome’s implications in host immunity [28,29], we investigated 22 KEGG immune pathways for enrichment with respect to these species. We would like to note that very few species resulted in a significant enrichment of these pathways. As such, we have not included these results in the current paper.

## 3. Discussion

Our results suggest that the tumoral microbiome is strongly reflective of CR in prostate cancer. Of all the cohorts, numerous species were found to be differentially abundant between the CRPC tumor and the normal tissue samples. Twelve of these species were common to all the cohorts. We believe that this may be attributed to castration itself, which has been shown to reduce the diversity in the gut microbiome [38,39]. Of these species, numerous were observed to correlate significantly to the expression of the chosen CR markers. AR expression was significantly greater in the samples with lesser abundance of *Veillonella parvula* and *Streptococcus pneumoniae*. The reverse was true of the genus *Staphylococcus*, which had previously been reported to be of greater abundance in prostate cancers [49]. Species of the genus *Shewanella* were also observed to correlate positively with AR expression and have been shown to be enriched in malignant prostate cancers [50]. The microbiome is known to be implicated in an array of human diseases and is thought to exert its effects through the release of metabolites [26,27,28,29]. It is unknown whether these metabolites interact directly with AR or its related proteins, though metabolomic analyses may speak to this influence.

PI3K-AKT signaling is also known to be upregulated in patients with CRPC [16]. We identified several PI3K and AKT family genes that correlated negatively to many of the species studied. The PI3K-AKT pathway as a whole was negatively enriched by many species, as well. The effects of probiotics on this pathway have been demonstrated [51]. It is thought that the metabolites of select species are capable of suppressing this pathway’s activation, ultimately suppressing a cancer’s growth [51]. Thus, decreased diversity in the microbiome, as observed in these patients with CRPC, may allow for the aberrant activation of the PI3K-AKT pathway. Further metabolomic analyses might confirm whether these two are indeed causally related. Nonetheless, the tumoral microbiome appears to closely follow the AR, PI3K-AKT, and endocrine resistance signaling pathways. Thus, dysbiosis of the microbiome may allow clinicians to monitor disease progression in patients with CRPC.

We also observed similar microbial relations to cancer stemness and pluripotency. Numerous CSC markers were found to correlate significantly to the abundance of these species, including *Brevundimonas subvibrioides* and *Geobacillus thermodenitrificans*. Among other markers, MYC, EGFR, and SLC3A2 were consistently downregulated, with a greater abundance of most species. Hence, a lesser abundance of these species, as observed in these patients with CRPC, may correlate with increased MYC, EGFR, and SLC3A2 expression, increased MYC, EGFR, and SLC3A2 signaling, and, ultimately, a CSC-like phenotype [43,44]. Interestingly, overexpression of EGFR has been implicated in the metastasis of prostate cancers to the bone [43]. SLC3A2 has similarly been shown to regulate proliferation, migration, and therapy resistance in cancer cells [44]. We propose that ablation of the microbiome, as induced by androgen deprivation therapy, may promote the growth and development of CSCs. In this way, the microbiome may mediate the rapid progression of CRPC. We note that this hypothesis is purely driven by the correlations above.

CSCs are thought to originate through the process of EMT as they migrate from the epithelium to the mesenchyme [19,20,21,23]. We found the EMT and pluripotency regulation pathways to be negatively enriched with respect to a majority of the species studied. Among the other genes in these pathways, the FGFR family genes and the Wnt family genes were consistently downregulated, with a greater abundance of these species. This was particularly true of the liver cohort. The Wnt signaling pathway is commonly aberrant in prostate cancers [52]. The FGFR family genes have been implicated in prostate cancers as contributors to metastasis [53,54]. Moreover, FGFR family genes are common to both the EMT and pluripotency regulation pathways. Our findings suggest that decreased microbial diversity in patients with CRPC, as observed, may correlate with an increased expression of these genes. Ultimately, this may result in the enrichment of the EMT pathway. The microbiome has been shown to regulate the process of EMT through metabolic interactions [46,47], though less is known of the microbiome’s relation to cellular pluripotency. Due to this, we suspect that the observed correlations of these species to the pluripotency regulation pathway may only be coincidental and mediated by EMT. Investigation of the interaction of microbial metabolites with the above CSC markers may be used to further test this hypothesis.

Ultimately, we found that the tumoral microbiome is highly reflective of CR and cancer stemness in prostate cancer. We must note that our results are limited, largely due to the correlational nature of the above analyses. Though we do not demonstrate a causal relationship between microbiome dysbiosis and CRPC, we hope that this study speaks to the relevance of the microbiome to this disease. Analysis of the metabolic interactions of these species may reveal whether the microbiome is of causal influence toward the acquisition of CR. Such research may also suggest the utility of targeting the microbiome as a therapeutic approach for CRPC. Relatedly, the procedures used to collect and sequence the samples of the above studies likely contained slight discrepancies. This has the potential to confound our conclusions, although we attempted to mitigate these differences using the normalization procedures described below. Lastly, we utilized a reference database of bacterial sequences to map the sequences of our samples. This will only capture the species that have been cultured on Earth, although this is a common limitation to all microbiome studies which utilize direct sequence alignment.

## 4. Materials and Methods

### 4.1. Data Acquisition

RNA sequencing data were downloaded from the dbGaP Data Browser (https://www.ncbi.nlm.nih.gov/gap/) for the bone and soft tissue biopsies of patients with metastatic CRPC (accessed on 24 December 2023). This high-throughput sequencing was performed using the Illumina HiSeq 2500 platform (Illumina, San Diego, CA, USA), which reports 99.9% accuracy in sequencing results. The samples spanned two studies: phs000915 (*n* = 147) and phs001141 (*n* = 143). Only bone (*n* = 159), lymph node (*n* = 92), and liver (*n* = 39) metastases were analyzed, as the remaining sites contained insufficient sample sizes (*n* < 30). Whole-exome sequencing data for adjacent normal biopsies were similarly downloaded from the above study, phs000915 (*n* = 84). This consisted of bone (*n* = 24), lymph node (*n* = 42), and liver (*n* = 18) samples, as well. Samples of each site were considered independently throughout the remainder of this study.

### 4.2. Bacterial Read Mapping

We mapped the above sequencing data to bacterial sequences using the software Pathoscope 2.0 [55]. The bacterial sequences were sourced from the NCBI Nucleotide Database (https://www.ncbi.nlm.nih.gov/nucleotide/) (accessed on 2 August 2023). This software attempts to map sequencing reads to a reference of human sequences. The software excludes these reads and then attempts to map the remaining reads to a reference of bacterial sequences.

### 4.3. Gene Read Mapping

We mapped the above sequencing data to the hg38 reference genome using the STAR 2.7.10a software [56]. The hg38 genome was sourced from the NCBI Nucleotide Database (https://www.ncbi.nlm.nih.gov/nucleotide/) (accessed on 2 August 2023). This was performed with the max number of mismatches set to 10, a mates max gap of 500,000, and a max multimapping of 10.

### 4.4. Cross-Study Normalization

Given that the above samples spanned two distinct studies, we chose to utilize cumulative sum scaling as a means of cross-study normalization. This technique divides the expression value of a gene by the sum of all genes’ expressions in that sample. The resultant expression values are relative and on scale of 0 to 1. By nature, the total expression of all the genes will be greater in some samples than others. This may present an issue when comparing the expression values of an individual gene between these samples, as any differences may purely be resultant of one of the samples being more transcriptionally active. With this technique, samples with a greater total expression of all genes are made more comparable to those with a lesser total expression of all genes. We similarly performed this technique on the samples’ species abundance counts. We utilized principle coordinate analysis (PCoA) to demonstrate the effectiveness of this technique. Sample dissimilarities were calculated by means of a Euclidean distance.

### 4.5. Microbial Contamination Correction

In tissue extraction and sequencing procedures, there remains the possibility for contaminant species to be introduced into a sample [37]. These species are not reflective of a patient’s tumoral microbiome and are likely introduced in a fixed amount. As such, these species are expected to be of similar abundances in all samples, regardless of the total abundance of taxa in a sample. To identify and exclude these species, the total abundance of all the species was tallied in each sample. Spearman’s correlations were used to assess the relationship of each individual species to the total abundance of all the species in each sample. Species that did not exhibit a significant relation (*p* < 0.05) were deemed contaminants and were excluded from further analyses.

### 4.6. Differential Abundance Analyses

The Kruskal–Wallis test was used to identify species that were differentially abundant between the CRPC and the normal samples (*p* < 0.05). Only these species were included in the remaining analyses for each respective cohort.

### 4.7. Expression Correlation Analyses

A list of genes known to be implicated in CR at a transcriptional level was collected from the literature [18]. A list of known CSC markers was similarly collected [42,43,44,45]. Two of these studies investigate CSC markers, with a particular emphasis on prostate cancer. The third investigates CSC markers across several cancer types, and the fourth investigates SLC3A2 as a CSC marker, which has been implicated in the prognosis of prostate cancer [57]. Spearman’s correlations were used to assess the relation of each species’ abundance to each gene’s expression.

### 4.8. Gene Set Enrichment Analyses

The clusterProfileR v4.6.2 R package was used to assess pathway enrichment with respect to each species’ abundance [58]. The pathways were sourced from the KEGG PATHWAY Database (https://www.genome.jp/kegg/pathway.html) (accessed on 28 December 2023). The prostate cancer (hsa05215), PI3K-AKT (hsa04151), and endocrine resistance (hsa01522) pathways were used to model CR. The adherens junction pathway (hsa04520) and the signaling pathway regulating the pluripotency of stem cells (hsa04550) were used to model EMT and stemness, respectively.

## 5. Conclusions

Our observations suggest that the microbiome is heavily associated with CR and stemness in prostate cancer. Numerous species were observed to correlate strongly with the expression of the CR and CSC markers studied. These species further correlated with the enrichment of select signaling pathways involved in CR and cancer stemness. The potential regulation of these factors by the microbiome should be further investigated to explore the microbiome’s relevance to CRPC. Ultimately, this may provide great nuance with respect to the aggressive nature of this disease. By exploring the microbiome’s implications in CR, stemness, and metastasis, we may better understand the pathology of high-risk prostate cancers, allowing for the needed improvements in the treatment of patients with these diseases.

## Data Availability

All the data can be access through the dbGaP Data Browser (https://www.ncbi.nlm.nih.gov/gap/) under accessions phs000915 and phs001141 (accessed on 24 December 2023).

## Supplementary Materials

The following supporting information can be downloaded at https://www.mdpi.com/article/10.3390/ijms25063291/s1.
