# Supplementary figures and images for: Microbiome Dysbiosis Is Associated with Castration Resistance and Cancer Stemness in Metastatic Prostate Cancer
Source: Int J Mol Sci. 2024 Mar 14;25(6):3291. doi: 10.3390/ijms25063291 (PMC10970102; doi:10.3390/ijms25063291)

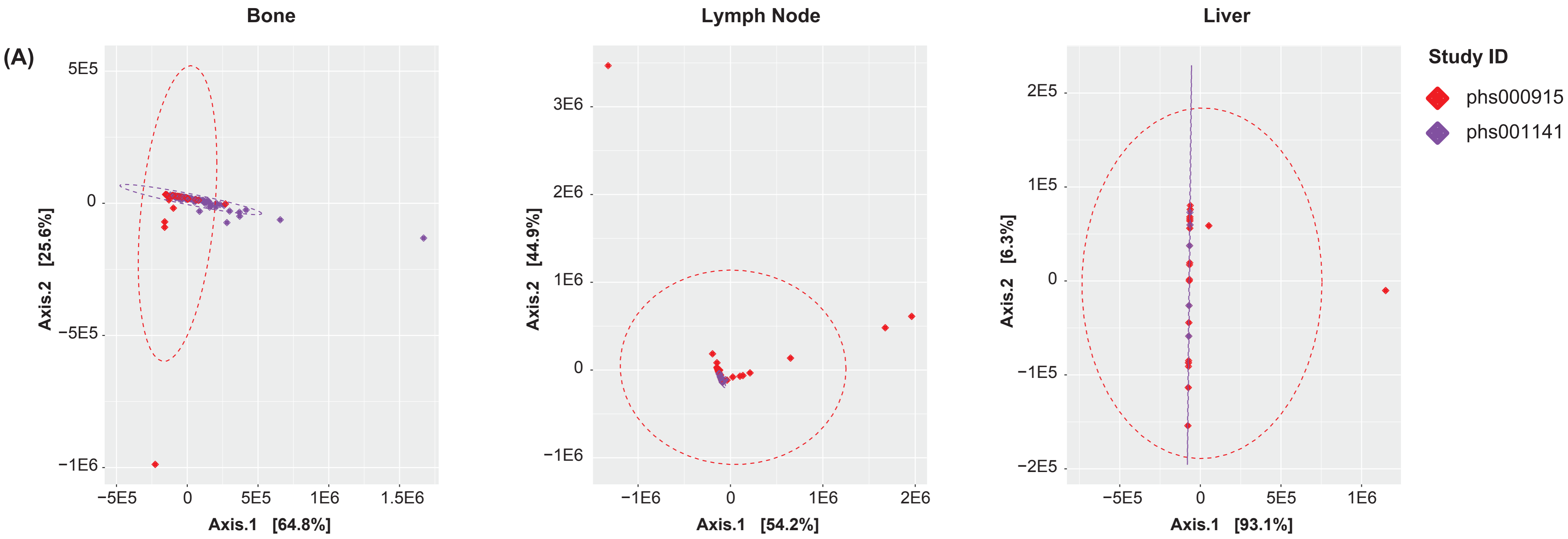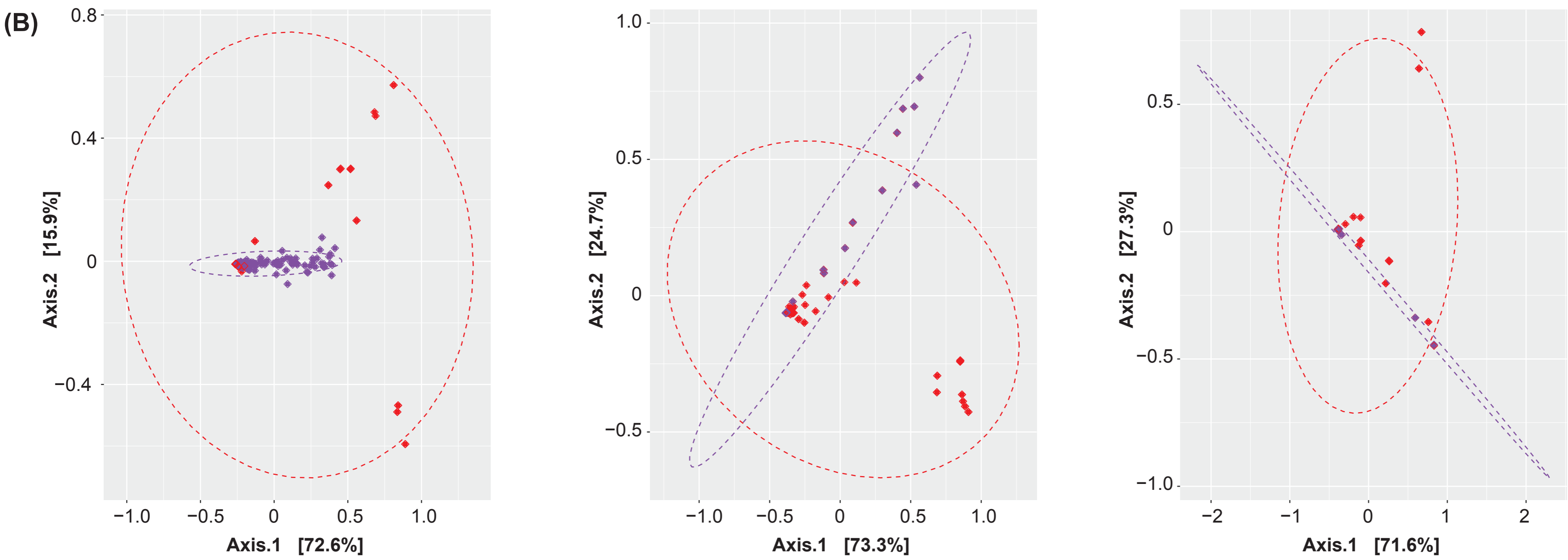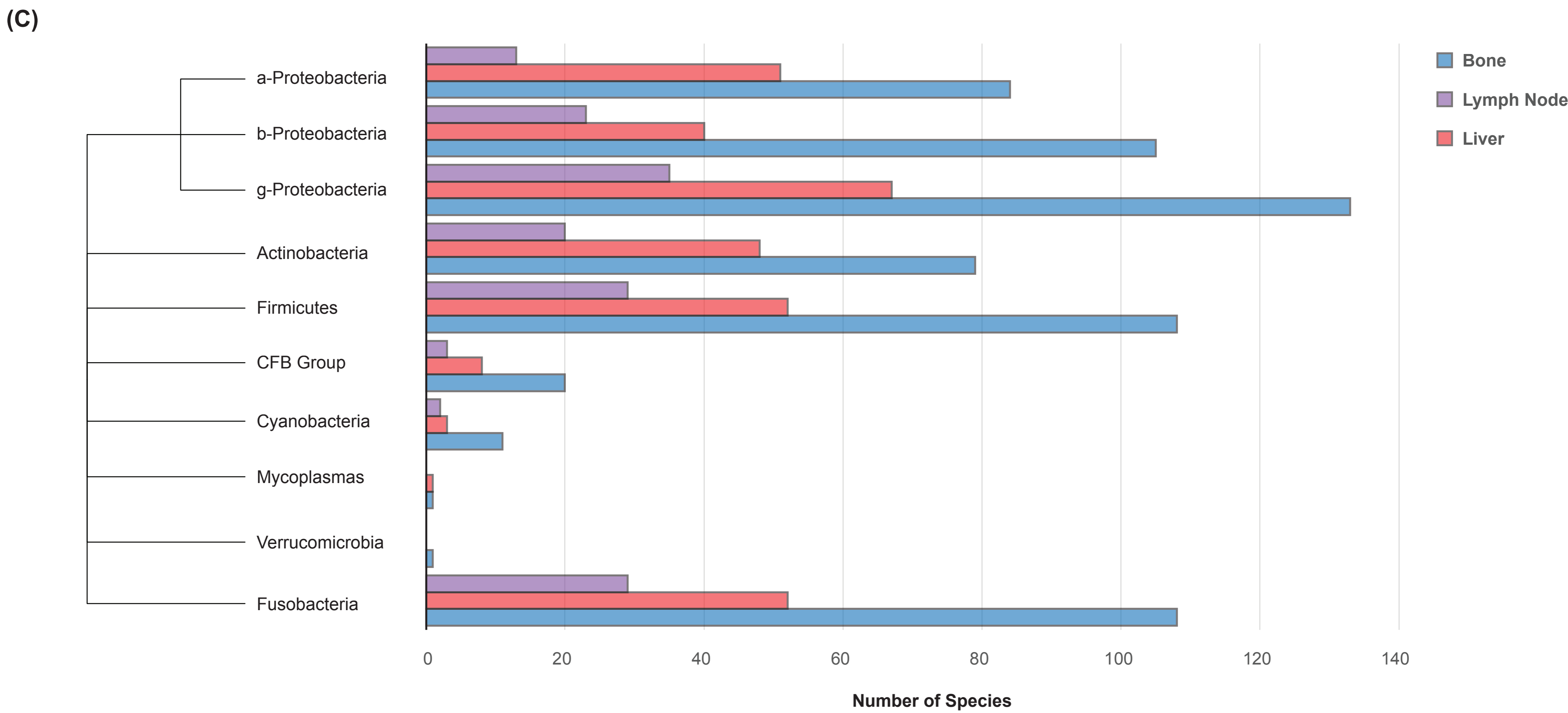

Supplement: Supplementary file 1 [file ijms-25-03291-s001.zip › Supplementary Figure S1.pdf]

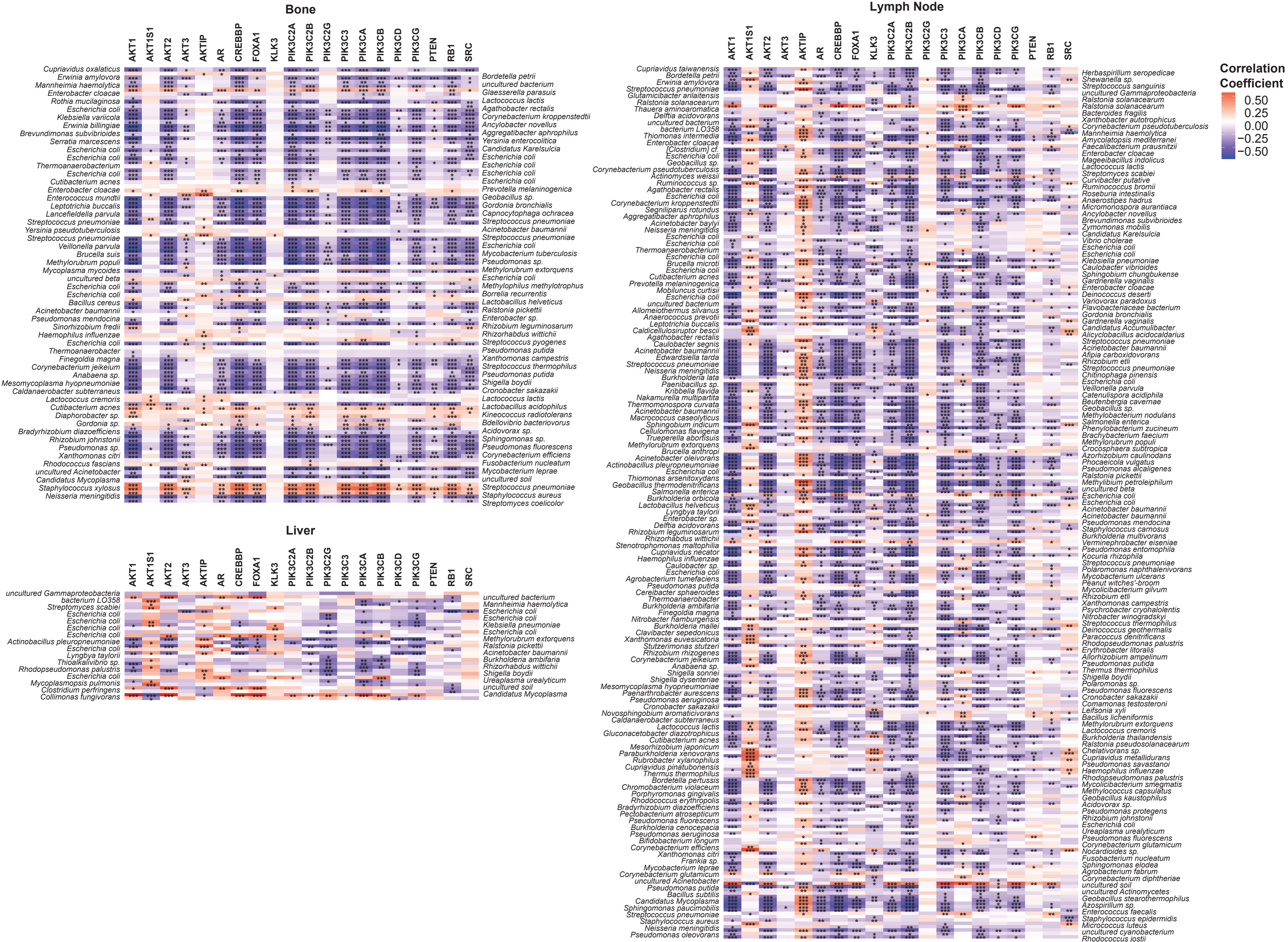

Supplement: Supplementary file 1 [file ijms-25-03291-s001.zip › Supplementary Figure S2.pdf]
