# Supplementary material for: Microbiome Dysbiosis Is Associated with Castration Resistance and Cancer Stemness in Metastatic Prostate Cancer
Source: Int J Mol Sci. 2024 Mar 14;25(6):3291. doi: 10.3390/ijms25063291 (PMC10970102; doi:10.3390/ijms25063291)
Supplement: Supplementary file 1 [file Supplementary Figure S3.pdf › Supplementary Figure S3.pdf]

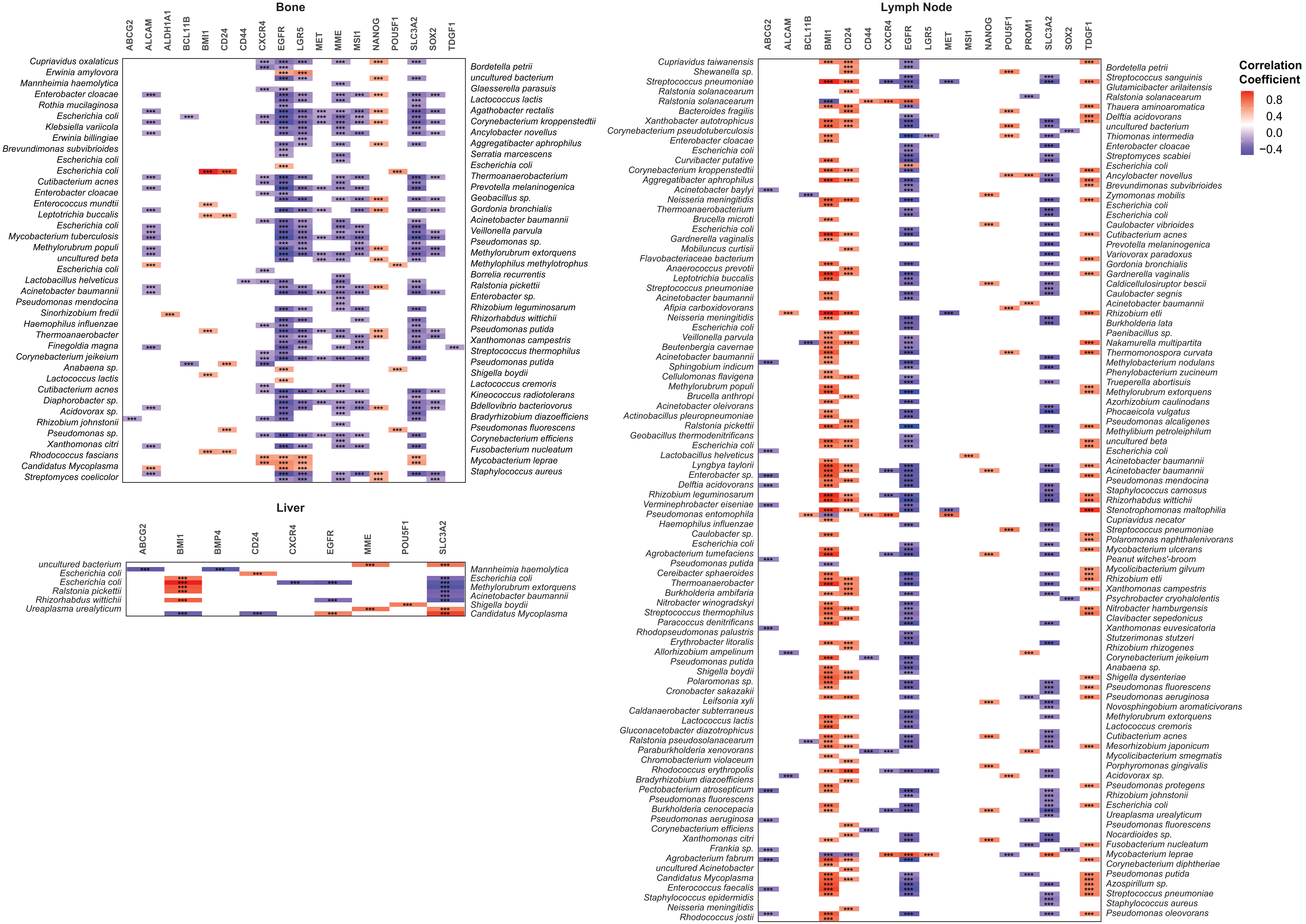
Bone
ABCG2
ALCAM
ALDH1A1
BCL11B
BMI1
CD24
CD44
CXCR4
EGFR
LGR5
MET
MME
MSI1
NANOG
POU5F1
SLC3A2
SOX2
TDGF1
Cupriavidus oxalaticus
Erwinia amylovora
Mannheimia haemolytica
Enterobacter cloacae
Rothia mucilaginosa
Escherichia coli
Klebsiella variicola
Erwinia billingiae
Brevundimonas subvibrioides
Escherichia coli
Escherichia coli
Cutibacterium acnes
Enterobacter cloacae
Enterococcus mundtii
Leptotrichia buccalis
Escherichia coli
Mycobacterium tuberculosis
Methylorubrum populi
uncultured beta
Escherichia coli
Lactobacillus helveticus
Acinetobacter baumannii
Pseudomonas mendocina
Sinorhizobium fredii
Haemophilus influenzae
Thermoanaerobacter
Finegoldia magna
Corynebacterium jeikeium
Anabaena sp.
Lactococcus lactis
Cutibacterium acnes
Diaphorobacter sp.
Acidovorax sp.
Rhizobium johnstonii
Pseudomonas sp.
Xanthomonas citri
Rhodococcus fascians
Candidatus Mycoplasma
Streptomyces coelicolor
Bordetella petrii
uncultured bacterium
Glaesserella parasuis
Lactococcus lactis
Agathobacter rectalis
Corynebacterium kroppenstedtii
Ancylobacter novellus
Aggregatibacter aphrophilus
Serratia marcescens
Escherichia coli
Thermoanaerobacterium
Prevotella melaninogenica
Geobacillus sp.
Gordonia bronchialis
Acinetobacter baumannii
Veillonella parvula
Pseudomonas sp.
Methylorubrum extorquens
Methylophilus methylotrophus
Borrelia recurrentis
Ralstonia pickettii
Enterobacter sp.
Rhizobium leguminosarum
Rhizorhabdus wittichii
Pseudomonas putida
Xanthomonas campestris
Streptococcus thermophilus
Pseudomonas putida
Shigella boydii
Lactococcus cremoris
Kineococcus radiotolerans
Bdellovibrio bacteriovorus
Bradyrhizobium diazoefficiens
Pseudomonas fluorescens
Corynebacterium efficiens
Fusobacterium nucleatum
Mycobacterium leprae
Staphylococcus aureus
Liver
ABCG2
BMI1
BMP4
CD24
CXCR4
EGFR
MME
POU5F1
SLC3A2
uncultured bacterium
Escherichia coli
Escherichia coli
Ralstonia pickettii
Rhizorhabdus wittichii
Ureaplasma urealyticum
Mannheimia haemolytica
Escherichia coli
Methylorubrum extorquens
Acinetobacter baumannii
Shigella boydii
Candidatus Mycoplasma
Lymph Node
ABCG2
ALCAM
BCL11B
BMI1
CD24
CD44
CXCR4
EGFR
LGR5
MET
MSI1
NANOG
POU5F1
PROM1
SLC3A2
SOX2
TDGF1
Cupriavidus taiwanensis
Shewanella sp.
Streptococcus pneumoniae
Ralstonia solanacearum
Ralstonia solanacearum
Bacteroides fragilis
Xanthobacter autotrophicus
Corynebacterium pseudotuberculosis
Enterobacter cloacae
Escherichia coli
Curvibacter putative
Corynebacterium kroppenstedtii
Aggregatibacter aphrophilus
Acinetobacter baylyi
Neisseria meningitidis
Thermoanaerobacterium
Brucella microti
Escherichia coli
Gardnerella vaginalis
Mobiluncus curtisii
Flavobacteriaceae bacterium
Anaerococcus prevotii
Leptotrichia buccalis
Streptococcus pneumoniae
Acinetobacter baumannii
Afipia carboxidovorans
Neisseria meningitidis
Escherichia coli
Veillonella parvula
Beutenbergia cavernae
Acinetobacter baumannii
Sphingobium indicum
Cellulomonas flavigena
Methylorubrum populi
Brucella anthropi
Acinetobacter oleivorans
Actinobacillus pleuropneumoniae
Ralstonia pickettii
Geobacillus thermodenitrificans
Escherichia coli
Lactobacillus helveticus
Lyngbya taylorii
Enterobacter sp.
Delftia acidovorans
Rhizobium leguminosarum
Verminephrobacter eiseniae
Pseudomonas entomophila
Haemophilus influenzae
Caulobacter sp.
Escherichia coli
Agrobacterium tumefaciens
Pseudomonas putida
Cereibacter sphaeroides
Thermoanaerobacter
Burkholderia ambifaria
Nitrobacter winogradskyi
Streptococcus thermophilus
Paracoccus denitrificans
Rhodopseudomonas palustris
Erythrobacter litoralis
Allorhizobium ampelinum
Pseudomonas putida
Shigella boydii
Polaromonas sp.
Cronobacter sakazakii
Leifsonia xyli
Caldanaerobacter subterraneus
Lactococcus lactis
Gluconacetobacter diazotrophicus
Ralstonia pseudosolanacearum
Paraburkholderia xenovorans
Chromobacterium violaceum
Rhodococcus erythropolis
Bradyrhizobium diazoefficiens
Pectobacterium atrosepticum
Pseudomonas fluorescens
Burkholderia cenocepacia
Pseudomonas aeruginosa
Corynebacterium efficiens
Xanthomonas citri
Frankia sp.
Agrobacterium fabrum
uncultured Acinetobacter
Candidatus Mycoplasma
Enterococcus faecalis
Staphylococcus epidermidis
Neisseria meningitidis
Rhodococcus jostii
Bordetella petrii
Streptococcus sanguinis
Glutamicibacter arilaitensis
Ralstonia solanacearum
Thauera aminoaromatica
Delftia acidovorans
uncultured bacterium
Thiomonas intermedia
Enterobacter cloacae
Streptomyces scabiei
Escherichia coli
Ancylobacter novellus
Brevundimonas subvibrioides
Zymomonas mobilis
Escherichia coli
Escherichia coli
Caulobacter vibrioides
Cutibacterium acnes
Prevotella melaninogenica
Variovorax paradoxus
Gordonia bronchialis
Gardnerella vaginalis
Caldicellulosiruptor bescii
Caulobacter segnis
Acinetobacter baumannii
Rhizobium etli
Burkholderia lata
Paenibacillus sp.
Nakamurella multipartita
Thermomonospora curvata
Methylobacterium nodulans
Phenylobacterium zucineum
Trueperella abortisuis
Methylorubrum extorquens
Azorhizobium caulinodans
Phocaeicola vulgatus
Pseudomonas alcaligenes
Methylibium petroleiphilum
uncultured beta
Escherichia coli
Acinetobacter baumannii
Acinetobacter baumannii
Pseudomonas mendocina
Staphylococcus carnosus
Rhizorhabdus wittichii
Stenotrophomonas maltophilia
Cupriavidus necator
Streptococcus pneumoniae
Polaromonas naphthalenivorans
Mycobacterium ulcerans
Peanut witches'-broom
Mycolicibacterium gilvum
Rhizobium etli
Xanthomonas campestris
Psychrobacter cryohalolentis
Nitrobacter hamburgensis
Clavibacter sepedonicus
Xanthomonas euvesicatoria
Stutzerimonas stutzeri
Rhizobium rhizogenes
Corynebacterium jeikeium
Anabaena sp.
Shigella dysenteriae
Pseudomonas fluorescens
Pseudomonas aeruginosa
Novosphingobium aromaticivorans
Methylorubrum extorquens
Lactococcus cremoris
Cutibacterium acnes
Mesorhizobium japonicum
Mycolicibacterium smegmatis
Porphyromonas gingivalis
Acidovorax sp.
Pseudomonas protegens
Rhizobium johnstonii
Escherichia coli
Ureaplasma urealyticum
Pseudomonas fluorescens
Nocardioides sp.
Fusobacterium nucleatum
Mycobacterium leprae
Corynebacterium diphtheriae
Pseudomonas putida
Azospirillum sp.
Streptococcus pneumoniae
Staphylococcus aureus
Pseudomonas oleovorans
Correlation Coefficient
0.8
0.4
0.0
−0.4
